# Trends in the Management of Bladder Cancer with Emphasis on Frailty: A Nationwide Analysis of More Than 49,000 Patients from a German Hospital Network

**DOI:** 10.3390/life16010169

**Published:** 2026-01-21

**Authors:** Tobias Klatte, Frederic Bold, Julius Dengler, Michela de Martino, Sven Hohenstein, Ralf Kuhlen, Andreas Bollmann, Thomas Steiner, Nora F. Dengler

**Affiliations:** 1 Department of Urology, Helios Klinikum Bad Saarow, 15526 Bad Saarow, Germany; 2 Faculty of Health Sciences, Brandenburg Medical School Theodor Fontane, 14770 Brandenburg, Germany; 3 Department of Anaesthesiology and Intensive Care Medicine, Helios Klinikum Bad Saarow, 15526 Bad Saarow, Germany; 4 Department of Neurosurgery, Helios Klinikum Bad Saarow, 15526 Bad Saarow, Germany; 5 Real World Evidence and Health Technology Assessment, Helios Health Institute, 13125 Berlin, Germany; sven.hohenstein@helios-gesundheit.de (S.H.);; 6 Department of Urology, Helios Klinikum Erfurt, 99089 Erfurt, Germany

**Keywords:** bladder cancer, frailty, prognosis, chemotherapy

## Abstract

Background: Bladder cancer (BC) predominantly affects older patients, and their multidisciplinary treatment often includes surgical intervention. Frailty can influence treatment decisions and is associated with poorer outcomes. This study analyses trends in demographics, treatment patterns and frailty in a large, nationwide, real-world inpatient cohort in Germany. Methods: This retrospective observational study included a total of 49,139 consecutive patients, who received inpatient care for BC at all HELIOS hospitals in Germany between 2016 and 2022. Frailty was assessed using the Hospital Frailty Risk Score (HFRS) and categorised as low (<5), intermediate (5–15), or high (>15). Trends in HFRS, treatment modalities, and demographic variables were analysed using regression models and compared between the periods 2016–2019 and 2020–2022. Results: Of the 49,139 patients, 27,979 were treated between 2016–2019 and 21,160 between 2020–2022. Patients treated in the later period were slightly older but had a lower comorbidity index. The proportion of patients with low frailty increased (73.4% vs. 75.5%, *p* < 0.01), intermediate frailty decreased (23.5% vs. 21.5%, *p* < 0.01) and the proportion of highly frail patients remained stable at 3.0% (*p* = 0.95). Rates of transurethral resection declined over time, whereas rates of RC remained stable (*p* = 0.12). The use of systemic therapy increased (*p* = 0.003), particularly among low frailty elderly patients. Early intravesical chemotherapy following transurethral resection declined significantly in 2020–2022 (*p* < 0.001), particularly among elderly patients with high frailty. Mean length of hospital stay decreased by one day, while ICU admission rates and in-hospital mortality remained stable across time periods. Conclusions: This study shows frailty-specific changes in hospitalisation patterns and inpatient management of BC in Germany, underscoring the value of frailty assessment in population-based research. The proportion of patients classified as having low frailty increased over time. Significant changes in the use of intravesical chemotherapy and systemic therapy were associated with frailty. The decline in early intravesical chemotherapy may have implications for recurrence risk and downstream healthcare utilisation.

## 1. Introduction

Bladder cancer (BC) is one of the most common malignancies worldwide, with an estimated 614,000 new cases and over 220,000 deaths annually [1]. It ranks as the ninth most common cancer [1], with urothelial carcinoma comprising more than 90% of cases [2]. BC is more common in men than in women and mainly affects individuals aged 65 and above, with a median age at diagnosis of approximately 73 years [3]. As life expectancy continues to increase, the annual incidence and mortality is expected to increase by more than 70% until 2040 [4]. Management pathways for BC range from transurethral resection (TURBT) with intravesical therapies in patients with non-muscle invasive BC to radical cystectomy (RC) and systemic therapies in more advanced stages [5,6,7,8,9]. The economic burden of BC is among the highest of all malignancies, primarily driven by frequent recurrences requiring further surgeries, lifelong surveillance, complex interventions and new targeted therapies [10]. 

Frailty as a clinical syndrome has gained increased attention as a critical predictor of outcomes among patients with BC [11,12]. Beyond chronological age, it captures multiple dimensions of function, medication, cognition, and nutrition, which may not be apparent through routine clinical assessment [11]. The prevalence of frailty in cancer patients may be as high as 40%, and its presence is associated with surgical outcomes, hospitalisation and mortality [13]. Despite its clinical relevance, frailty remains under-assessed and inconsistently documented in many healthcare settings. Administrative tools, such as the Hospital Frailty Risk Score (HFRS) [14], offer scalable and reproducible means to identify frail individuals using routinely collected data and facilitate large-scale population-based analyses [15,16]. 

In the context of BC, frailty poses unique challenges [11]. Patients with muscle-invasive disease may be candidates for complex radical surgery, but this has a considerable risk of complications, death, readmission, and prolonged recovery [6]. Even non-muscle-invasive BC requires intensive, invasive monitoring through cystoscopy, repeated TURBTs under spinal or general anaesthetic and intravesical treatments [5], placing considerable strain on patients. Prior studies have demonstrated that frailty is associated with worse perioperative outcomes, prolonged hospitalisation, reduced quality of life, and impaired overall survival [17,18,19]. However, many studies are limited by small sample sizes, single-centre cohorts, or reliance on measures of frailty that are not routinely available in administrative datasets. Furthermore, there are no comprehensive investigations on frailty among BC patients within the German hospital system.

In this study, we aim to address these gaps by utilising data from the HELIOS hospital network, Germany’s largest private hospital provider. We analysed trends in frailty among patients hospitalised with BC between 2017 and 2022, using the validated HFRS. We examined the temporal evolution of frailty, treatment patterns and in-hospital mortality, aiming to generate real-world insights into BC care within the German healthcare system.

## 2. Methods

### 2.1. Study Design

Real world data from the administration of a nationwide network of 76 hospitals in Germany were retrospectively examined for this observational study. Of all national inpatient cases, 7% are managed within the HELIOS hospital network. The Hospital network covers urban and rural areas in 13 of the 16 federal states in Germany. In this study, patients admitted for malignant neoplasms of the bladder with the International Statistical Classification of Diseases and Related Health Problems (ICD-10) C67 that were treated between 2016 and 2022 were included. To identify possible differences in admission policies and treatments, the observation period was divided into 2016–2019 and 2020–2022. No exclusion criteria were defined. The nationwide standards for “Operations and Procedures codes” were used to classify treatment strategies and analysed for differences between the two time periods. Ethical approval was granted by the Ethics Committee of the University of Leipzig (protocol registration number 490/20-ek, date of approval 7 October 2022).

### 2.2. Assessment of Frailty

Frailty was assessed using the validated HFRS [14]. The HFRS allows for systematic analysis and quantification of frailty through a total of 109 co-morbidities. Relevant ICD-10 codes are assigned points with varying weights, and the cumulative score is categorised according to three levels of frailty: low (HFRS < 5 points), intermediate (HFRS 5–15 points), and high (HFRS > 15 points) frailty [14]. Although HRFS was primarily validated in patients aged ≥ 75 years, this score has been widely used in analyses of administrative datasets independent of age [15,16,20]. Additionally, recent data show that it is predictive of important clinical outcomes across all age categories [21,22]. Further details about HFRS are included in the Supplementary Material.

### 2.3. Sex, Age and Comorbidities

To analyse for the possible confounding factors sex, age and comorbidities, a separate analysis for the respective cohorts were performed. Sex was dichotomised into male and female. Age groups were classified into a group < 44 years, a group between 45 and 54 years, a group between 55–64 years, a group between 65–74 years, a group between 75 and 84 years and a group ≥ 85 years. Secondary, a dichotomisation for elderly (≥65 years) and non-elderly patients (<64 years) was performed. Comorbidities were assessed via the Elixhauser Comorbidity Index, a tool that allows for quantification of burden of comorbidities [23,24].

### 2.4. Statistical Analysis

Administrative data were extracted from QlikView (QlikTech, Wayne, PA, USA). For the description of the patient characteristics of the cohorts, we employed *χ*^2^ tests for binary variables and analysis of variance for numeric variables. For the comparison of proportions of selected treatments and outcomes in the different cohorts, we used generalised linear mixed models with logit link function. Inferential statistics were based on generalised linear mixed models with hospitals as random factor [25]. Effects were estimated with the lme4 package (version 1.1-21) [26] in the R environment for statistical computing (version 4.0.2). Frailty scores were analysed with negative binomial models. For the analysis, we multiplied the scores with ten in order to achieve integer values. For all tests, we apply a two-tailed 5% error criterion for significance.

## 3. Results

### 3.1. Study Population

A total of 49,139 patients with BC were hospitalised and treated across the HELIOS hospital network in Germany between 2016 and 2022. Of these, 27,979 patients were treated from 2016 to 2019 and 21,160 patients were treated from 2020 to 2022. The mean number of BC patients treated annually was 7020 (SD, 67) and ranged from 6927 to 7118. Demographical characteristics of the patients showed slight changes over time (Table 1). The mean age across the cohort was 72 years, with a statistically significant albeit slight increase from 71.9 (SD, 11.0) in 2016–2019 to 72.4 (SD, 10.6) in 2020–2022 (*p* < 0.01). The sex distribution remained unchanged, with 77.3% male patients compared to 77.5% (*p =* 0.49). There were changes in the co-morbidity profile that led a decrease in the mean Elixhauser Comorbidity Index from 12.7 (SD, 9.8) to 12.0 (SD, 9.3) (*p* < 0.01). Metastatic disease was present in 6060 patients (12.3%), with a slight decrease from 12.7% in 2016–2019 to 11.8% in 2020–2022 (*p* < 0.01).

**Figure 1:**
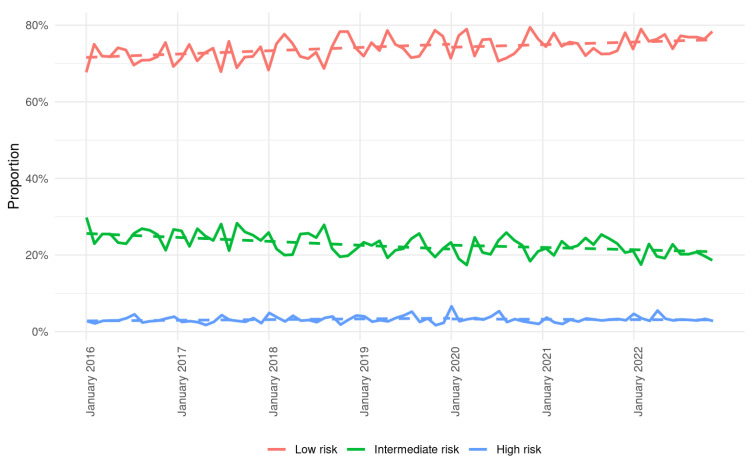
Changes in Hospital Frailty Risk Score over time. There was a continuous increase in patients with low frailty risk (<5 points) and a continuous decrease in patients with intermediate frailty risk (5–15 points). The proportion of patients with high frailty risk (>15 points) remained stable.

Frailty, according to HFRS, showed a significant shift from the former to the later period (Table 1). Indeed, the proportion of patients with low frailty increased from 73.4% in 2016–2019 to 75.5% in 2020–2022 (*p* < 0.01). Conversely, the proportion of patients with intermediate frailty decreased from 23.5% to 21.5% (*p* < 0.01). There was a stable proportion of highly frail patients at 3.0% in both time periods (*p =* 0.95) (Figure 1).

### 3.2. Trends in Treatments and Outcomes

Results are summarised in Table 2. There was a significant decrease in rates of TURBT from 72.9% to 70.8% (*p* < 0.001), while the rates of RC showed no change (5.6% vs. 5.9%, *p* = 0.12). The vast majority of RCs were performed via the open approach, although the proportion of robotic-assistant RC increased 15-fold during 2020–2022 (*p* < 0.001). However, frailty risk profiles did not change in both treatment categories.

Consistent with the increase in advanced disease, there was a significant increase in the proportion of patients receiving systemic therapy (from 9.7% to 10.4%, *p* = 0.003) and best supportive care (from 0.7% to 1.3%, *p* < 0.001). In low frailty patients, the proportion of patients who received systemic therapy increased from 10.2% in 2016–2019 to 11.2% in 2020–2022 (*p* = 0.002). This increase was most pronounced in elderly patients (*p* = 0.001, Table 3). In contrast, there were no changes in intermediate (*p* = 0.40) and high frailty patients (*p* = 0.56, Table 3). The use of in-hospital best supportive care increased in both elderly and non-elderly patients, but was independent from the frailty risk profile (each *p* > 0.2).

Early intravesical chemotherapy instillations following TURBT showed a significant decline from 19.9% 2016–2019 to 17.3% in 2020–2022 (*p* < 0.001, Table 4). Here, we observed a significant association with age and frailty. In low frailty patients, the decline in the use of early instillation was seen in both non-elderly and elderly patients (each *p* < 0.001), but it was greater in the former group (4.4% vs. 2.7%). In intermediate frailty patients, the likelihood of an early instillation even increased from 18.5% to 19.3% in non-elderly patients (*p* < 0.001), while it remained stable in the elderly (*p* = 0.65). The decline in early instillation was most pronounced in elderly patients with high frailty. Indeed, its use decreased from 11.3% in 2016–2019 to 4.3% in 2020–2022 (*p* = 0.008).

There were no changes in ICU admission rates (*p* = 0.23). Length of stay decreased by a mean/median of 1 day (*p* < 0.001). In-hospital mortality remained stable in the two time periods, and was 2.3% in 2016–2019 and 2.6% in 2020–2022 (*p* = 0.06). Mortality rates also did not vary significantly by frailty category across time.

## 4. Discussion

Our data suggest that the frailty profile of hospitalised BC patients changed between 2016 and 2022, with a higher proportion of patients classified as low frailty in the later period. Rates of TURBT declined over time, whereas rates of RC remained stable and the use of systemic therapy increased. Importantly, the application of early intravesical chemotherapy following TURBT decreased, with the most pronounced decline observed among elderly patients with high frailty.

Our findings confirm that a substantial proportion of patients hospitalised for BC care exhibit intermediate or high frailty risk, underscoring the clinical relevance of frailty assessment in this population. A recent review by Parikh and Sharma highlighted frailty as a valuable tool for preoperative planning and clinical decision-making in BC patients [27]. Our real-world data indicate that the frailty risk profile of hospitalised BC patients shifted toward lower frailty. Given the fact that patients were slightly older in the later period, this pattern suggests a potential selection effect, whereby more frail patients may have been less likely to be admitted or to undergo hospital-based interventions. Similar selection phenomena were reported during the pandemic, particularly among older, frail patients with advanced disease or poor performance status, for whom oncological treatments were frequently postponed [28]. Although the pandemic may have contributed to changes in frailty profiles, similar trends have been reported in both malignant and benign conditions, suggesting a more general phenomenon rather than a BC-specific effect [15,16,20]. This supports the assumption that the observed shift toward lower frailty reflects true changes in the hospitalised population rather than artefacts of coding or case mix.

Notably, rates of TURBT and early intravesical chemotherapy after TURBT declined, while RC rates remained unchanged. The reduction in TURBT was not associated with age or frailty, whereas the decline in early intravesical chemotherapy was most pronounced among elderly patients with high frailty. The reasons for the observed decline remain speculative, but may reflect attempts to minimise hospital stay duration and treatment-related risks, particularly during the COVID-19 pandemic. Nevertheless, our findings clearly demonstrate that, in real-world clinical practice, age and frailty substantially influence the decision to administer early intravesical chemotherapy. While a recent survey highlighted that such decisions are primarily guided by institutional protocols and tumour-related factors [29], population-based analyses have revealed marked disparities based on patient-specific characteristics, including lower utilisation of intravesical chemotherapy in older patients [30]. Our data are in line with these findings.

The observed decline in early intravesical chemotherapy may have relevant clinical implications. In an individual patient data meta-analysis, early intravesical chemotherapy decreased the odds of recurrence by 35% compared to TURBT alone [31]. The observed decrease in the use of early instillations will therefore likely increase recurrence rates, resulting in a greater need for repeat TURBTs, with associated risks of complications, additional intravesical treatments, increased downstream healthcare utilization, and higher costs. Accordingly, our findings suggest that treatment de-escalation in a potentially vulnerable subgroup of elderly patients with high frailty may carry clinically meaningful consequences.

Robot-assisted RC has been shown to be non-inferior to open surgery, with potential benefits such as reduced blood loss and shorter hospital stays [32,33]. In our cohort, robot-assisted surgery was used in only a small proportion of patients, but its adoption increased in the later period. This trend likely reflects the introduction of robotic platforms within the HELIOS hospital network starting in 2019, followed by a progressive expansion of robotic RC programmes.

We observed an increase in systemic therapy use, particularly among elderly patients with low frailty. This finding should be interpreted cautiously, as our dataset does not differentiate between neoadjuvant, adjuvant, and palliative indications. There are also no data on the agents used for systemic therapy. The increase may reflect a broader adoption of neoadjuvant protocols, which historically has been underutilised in Germany [34]. Additionally, rates of in-hospital best supportive care, although low overall, increased in both elderly and non-elderly patients over time. This underscores the persistent underutilisation of best supportive care, a pattern that has been reported in previous research [35].

It is important to distinguish between frailty and comorbidity [36], particularly in analyses based on administrative datasets such as in our study. While some overlap is inevitable, the Elixhauser Comorbidity Index [23,24] primarily reflects disease burden, whereas the HFRS [14] captures a broader construct of vulnerability, including functional impairment, cognitive disorders, and geriatric syndromes. The concurrent use of both measures allowed us to partially disentangle these complementary dimensions of patient risk.

Despite changes in frailty distribution and treatment patterns, in-hospital mortality remained stable across time periods and frailty categories, suggesting that treatment decisions were likely adapted appropriately to patient risk profiles. The overall in-hospital mortality rate was relatively low, which may partly be explained by the heterogeneity of interventions and the fact that not all patients underwent high-risk procedures. The observed reduction in length of stay aligns with broader trends in the German healthcare system toward shorter hospitalisations [20].

The major strength of this study lies in the provision of large-scale, multicentre, frailty-specific real-world data on BC care in Germany. However, a number of important limitations inherent to the use of administrative inpatient data warrant careful consideration. First, our dataset lacks granular oncological information, including tumour stage (e.g., TNM classification), risk stratification, and molecular or histological details. As a result, we were unable to adjust analyses for disease severity beyond broad proxies such as metastatic coding, nor could we differentiate between non-muscle-invasive and muscle-invasive disease with sufficient precision. Similarly, systemic therapies could only be identified at a procedural level, without information on specific agents, treatment intensity, or treatment intent. Consequently, we were unable to distinguish between neoadjuvant, adjuvant, and palliative indications, which limits the interpretation of observed increases in systemic therapy use over time. The trends may therefore reflect shifts in treatment indications, evolving guidelines, or changes in case mix rather than true intensification of oncological therapy. Linkage of administrative data with clinical cancer registries would provide these critical variables and enable more refined oncological analyses, but was not feasible within the current data governance framework. Second, the observation period from 2020 to 2022 coincided with the COVID-19 pandemic, which substantially altered hospital admission patterns, resource availability, and treatment prioritisation. Frail, elderly, or advanced-stage patients may have been selectively deferred from hospital-based interventions, potentially contributing to the observed shift toward lower frailty among hospitalised patients. Although similar trends have been reported across other disease entities, residual pandemic-related confounding cannot be excluded. Third, our analysis is limited to in-hospital care and short-term outcomes. Long-term endpoints such as overall survival, cancer-specific survival, recurrence, progression, and post-discharge quality of life or frailty were not captured in our administrative inpatient dataset. Therefore, our findings should be interpreted as a snapshot of real-world inpatient management rather than a comprehensive assessment of oncological effectiveness or patient-centred outcomes. Finally, while the HFRS is a validated and scalable tool for population-based research, it remains an indirect measure of frailty derived from diagnostic coding and may incompletely capture functional, cognitive, and social dimensions of vulnerability. While the HFRS was applied across all age groups as in other studies [21,22], it has been formally validated only in patients aged ≥ 75 years. Consequently, frailty estimates in younger patients may partly reflect comorbidity burden rather than frailty in its strict geriatric sense. 

Despite these limitations, this nationwide real-world analysis of more than 49,000 BC patients demonstrates frailty-specific changes in hospitalisation patterns and treatment strategies in Germany. Our findings highlight the growing importance of frailty as a determinant of real-world clinical decision-making and provide a contemporary snapshot of frailty-adapted BC care.

## Figures and Tables

**Table 1:** Patient demographics, frailty and clinical characteristics by time period.

	2016–2019	2020–2022	*p* Value
Number of cases	27,979	21,160	
Age			
Mean (SD)	71.9 ± 11.0	72.4 ± 10.6	<0.01
≤44 years	1.3% (367)	1.1% (228)	0.02
45−54 years	5.4% (1507)	3.8% (809)	<0.01
55−64 years	18.0% (5045)	18.7% (3953)	0.07
65−74 years	27.7% (7747)	30.2% (6380)	<0.01
75−84 years	37.1% (10,394)	34.9% (7378)	<0.01
≥85 years	10.4% (2919)	11.4% (2412)	<0.01
Sex			
Male	77.3% (21,613)	77.5% (16,403)	
Female	22.7% (6364)	22.5% (4757)	0.49
Hospital Frailty Risk Score			
Low risk	73.4% (20,549)	75.5% (15,973)	<0.01
Intermediate risk	23.5% (6589)	21.5% (4548)	<0.01
High risk	3.0% (841)	3.0% (639)	0.95
Elixhauser comorbidity index			
Mean (SD)	12.7 ± 9.8	12.0 ± 9.3	<0.01
0	0.0% (12)	0.0% (7)	0.75
1–4	7.8% (2195)	7.6% (1617)	0.41
≥5	91.7% (25,661)	91.9% (19,441)	0.53
Comorbidities			
Congestive heart failure	9.9% (2769)	9.1% (1934)	<0.01
Cardiac arrhythmia	16.2% (4535)	15.3% (3227)	<0.01
Heart valve diseases	4.2% (1175)	3.8% (800)	0.02
Peripheral vascular disease	10.8% (3027)	9.4% (1991)	<0.01
Hypertension, uncomplicated	57.1% (15,976)	55.0% (11,641)	<0.01
Hypertension, complicated	6.7% (1882)	9.6% (2032)	<0.01
Chronic pulmonary disease	9.2% (2581)	9.0% (1910)	0.46
Diabetes, uncomplicated	15.1% (4221)	15.9% (3365)	0.01
Diabetes, complicated	8.3% (2323)	8.3% (1756)	1
Hypothyroidism	8.1% (2274)	9.2% (1951)	<0.01
Renal failure	31.7% (8865)	28.7% (6068)	<0.01
Obesity	12.5% (3486)	10.8% (2293)	<0.01
Fluid and electrolyte disorders	9.8% (2740)	9.0% (1901)	<0.01
Depression	3.0% (833)	3.1% (665)	0.3
Metastatic disease	12.7% (3563)	11.8% (2497)	<0.01

**Table 2:** Treatment modalities, admission to ICU and in-hospital mortality by time period.

	2016–2019	2020–2022	Odds Ratio (95% CI)	*p* Value
Radical cystectomy	5.6% (1556)	5.9% (1257)	1.06 (0.98–1.15)	0.12
Robotic surgery *	0.1% (18)	1.5% (191)	15.3 (9.38–25.0)	<0.001
Transurethral resection	72.9% (20,383)	70.8% (14,980)	0.89 (0.85–0.92)	<0.001
Early instillation of chemotherapy †	19.9% (4062)	17.3% (2595)	0.90 (0.85–0.95)	<0.001
Systemic therapy	9.7% (2713)	10.4% (2202)	1.10 (1.03–1.17)	0.003
Best supportive care	0.7% (198)	1.3% (265)	1.93 (1.58–2.35)	<0.001
Insertion of nephrostomy	10.9% (3052)	9.5% (2020)	0.92 (0.86–0.98)	0.01
Admission to intensive care	7.7% (2162)	7.3% (1552)	0.96 (0.89–1.03)	0.23
In-hospital mortality	2.3% (648)	2.6% (537)	1.12 (0.99–1.26)	0.06
Length of stay Mean (SD) Median (IQR)	5.1 (6.8) 3.0 (2–5)	4.5 (6.1) 2.0 (2–4)	- -	<0.01

* amongst patients treated with RC. † amongst patients treated with TURBT.

**Table 3:** Early instillation of systemic therapy by frailty and age group.

Age	Frailty Group	2016–2019	2020–2022	*p* Value
All	Low, % (*n*)	10.2% (2101)	11.2% (1795)	0.002
	Intermediate, % (*n*)	8.9% (585)	8.4% (382)	0.40
	High, % (*n*)	3.2% (27)	3.9% (25)	0.56
Non-elderly (<65 years)	Low, % (*n*)	15.5% (885)	16.7% (688)	0.11
	Intermediate, % (*n*)	15.6% (175)	18.6% (149)	0.09
	High, % (*n*)	3.9% (3)	12.3% (9)	0.11
Elderly (≥65 years)	Low, % (*n*)	8.2% (1216)	9.3% (1107)	0.001
	Intermediate, % (*n*)	7.5% (410)	6.2% (233)	0.02
	High, % (*n*)	3.1% (24)	2.8% (16)	0.87

**Table 4:** Early instillation of intravesical chemotherapy by frailty and age group.

Age	Frailty Group	2016–2019	2020–2022	*p* Value
All	Low, % (*n*)	21.1% (3405)	17.9% (2198)	<0.001
	Intermediate, % (*n*)	15.9% (621)	15.5% (388)	0.72
	High, % (*n*)	10.8% (37)	4.0 (9)	0.006
Non-elderly (<65 years)	Low, % (*n*)	24.6% (1034)	20.2% (601)	<0.001
	Intermediate, % (*n*)	18.5% (94)	19.3% (58)	0.006
	High, % (*n*)	0% (0)	0% (0)	1
Elderly (≥65 years)	Low, % (*n*)	19.9% (2370)	17.2% (1597)	<0.001
	Intermediate, % (*n*)	15.5% (527)	15.0% (330)	0.65
	High, % (*n*)	11.3% (37)	4.3% (9)	0.008

## Data Availability

The data presented in this study are available on request from the corresponding author due to proprietary and contractual restrictions from Helios Healthcare.

## Supplementary Materials

The following supporting information can be downloaded at: https://www.mdpi.com/article/10.3390/life16010169/s1, File S1: Hospital Frailty Risk Score.
