# Supplementary material for: Trends in the Management of Bladder Cancer with Emphasis on Frailty: A Nationwide Analysis of More Than 49,000 Patients from a German Hospital Network
Source: Life (Basel). 2026 Jan 21;16(1):169. doi: 10.3390/life16010169 (PMC12843476; doi:10.3390/life16010169)
Supplement: Supplementary file 1 [file life-16-00169-s001.zip › life-4096417-supplementary.pdf]

Supplementary Material

Hospital Frailty Risk Score

The Hospital Frailty Risk Score [14] provides hospitals and health systems with a low-cost, systematic way to screen for frailty and identify a group of patients who are at greater risk of adverse outcomes and for whom a frailty-attuned approach might be useful. Technically, the score is weighted sum of 109 comorbidities, defined as three-digit ICD codes. We distinguished three risk groups based on the score: low risk (score < 5), intermediate risk (score 5–15), and high risk (score > 15). The following table provides an overview of the comorbidities together with their ICD codes used to create the hospital frailty risk score.

ICD	Disease	Weight
F00	Dementia in Alzheimer's disease	7.1
G81	Hemiplegia	4.4
G30	Alzheimer's disease	4
I69	Sequelae of cerebrovascular disease (secondary codes)	3.7
R29	Other symptoms and signs involving the nervous and musculoskeletal systems (R29.6 Tendency to fall)	3.6
N39	Other disorders of urinary system (includes urinary tract infection and urinary incontinence)	3.2
F05	Delirium, not induced by alcohol and other psychoactive substances	3.2
W19	Unspecified fall	3.2
S00	Superficial injury of head	3.2
R31	Unspecified haematuria	3
B96	Other bacterial agents as the cause of diseases classified to other chapters (secondary code)	2.9
R41	Other symptoms and signs involving cognitive functions and awareness	2.7
R26	Abnormalities of gait and mobility	2.6
I67	Other cerebrovascular diseases	2.6
R56	Convulsions, not elsewhere classified	2.6
R40	Somnolence, stupor and coma	2.5
T83	Complications of genitourinary prosthetic devices, implants and grafts	2.4
S06	Intracranial injury	2.4
S42	Fracture of shoulder and upper arm	2.3
E87	Other disorders of fluid, electrolyte and acid-base balance	2.3
M25	Other joint disorders, not elsewhere classified	2.3
E86	Volume depletion	2.3
R54	Senility	2.2
Z50	Care involving use of rehabilitation procedures	2.1
F03	Unspecified dementia	2.1
W18	Other fall on same level	2.1
Z75	Problems related to medical facilities and other health care	2
F01	Vascular dementia	2
S80	Superficial injury of lower leg	2
L03	Cellulitis	2
H54	Blindness and low vision	1.9
E53	Deficiency of other B group vitamins	1.9
Z60	Problems related to social environment	1.8
G20	Parkinson's disease	1.8
R55	Syncope and collapse	1.8
S22	Fracture of rib(s), sternum and thoracic spine	1.8
K59	Other functional intestinal disorders	1.8
N17	Acute renal failure	1.8
L89	Decubitus ulcer	1.7
Z22	Carrier of infectious disease	1.7
B95	Streptococcus and staphylococcus as the cause of diseases classified to other chapters	1.7
L97	Ulcer of lower limb, not elsewhere classified	1.6
R44	Other symptoms and signs involving general sensations and perceptions	1.6
K26	Duodenal ulcer	1.6

I95	Hypotension	1.6
N19	Unspecified renal failure	1.6
A41	Other septicaemia	1.6
Z87	Personal history of other diseases and conditions	1.5
J96	Respiratory failure, not elsewhere classified	1.5
X59	Exposure to unspecified factor	1.5
M19	Other arthrosis	1.5
G40	Epilepsy	1.5
M81	Osteoporosis without pathological fracture	1.4
S72	Fracture of femur	1.4
S32	Fracture of lumbar spine and pelvis	1.4
E16	Other disorders of pancreatic internal secretion	1.4
R94	Abnormal results of function studies	1.4
N18	Chronic renal failure	1.4
R33	Retention of urine	1.3
R69	Unknown and unspecified causes of morbidity	1.3
N28	Other disorders of kidney and ureter, not elsewhere classified	1.3
R32	Unspecified urinary incontinence	1.2
G31	Other degenerative diseases of nervous system, not elsewhere classified	1.2
Y95	Nosocomial condition	1.2
S09	Other and unspecified injuries of head	1.2
R45	Symptoms and signs involving emotional state	1.2
G45	Transient cerebral ischaemic attacks and related syndromes	1.2
Z74	Problems related to care-provider dependency	1.1
M79	Other soft tissue disorders, not elsewhere classified	1.1
W06	Fall involving bed	1.1
S01	Open wound of head	1.1
A04	Other bacterial intestinal infections	1.1
A09	Diarrhoea and gastroenteritis of presumed infectious origin	1.1
J18	Pneumonia, organism unspecified	1.1
J69	Pneumonitis due to solids and liquids	1
R47	Speech disturbances, not elsewhere classified	1
E55	Vitamin D deficiency	1
Z93	Artificial opening status	1
R02	Gangrene, not elsewhere classified	1
R63	Symptoms and signs concerning food and fluid intake	0.9
H91	Other hearing loss	0.9
W10	Fall on and from stairs and steps	0.9
W01	Fall on same level from slipping, tripping and stumbling	0.9
E05	Thyrotoxicosis [hyperthyroidism]	0.9
M41	Scoliosis	0.9
R13	Dysphagia	0.8
Z99	Dependence on enabling machines and devices	0.8
U80	Agent resistant to penicillin and related antibiotics	0.8
M80	Osteoporosis with pathological fracture	0.8
K92	Other diseases of digestive system	0.8
I63	Cerebral Infarction	0.8
N20	Calculus of kidney and ureter	0.7
F10	Mental and behavioural disorders due to use of alcohol	0.7
Y84	Other medical procedures as the cause of abnormal reaction of the patient	0.7
R00	Abnormalities of heart beat	0.7
J22	Unspecified acute lower respiratory infection	0.7
Z73	Problems related to life-management difficulty	0.6
R79	Other abnormal findings of blood chemistry	0.6
Z91	Personal history of risk-factors, not elsewhere classified	0.5
S51	Open wound of forearm	0.5
F32	Depressive episode	0.5
M48	Spinal stenosis (secondary code only)	0.5
E83	Disorders of mineral metabolism	0.4

M15	Polyarthrosis	0.4
D64	Other anaemias	0.4
L08	Other local infections of skin and subcutaneous tissue	0.4
R11	Nausea and vomiting	0.3
K52	Other noninfective gastroenteritis and colitis	0.3
R50	Fever of unknown origin	0.1
